# Heavy Metals and Metalloids as Autophagy Inducing Agents: Focus on Cadmium and Arsenic 

**DOI:** 10.3390/cells1030597

**Published:** 2012-08-27

**Authors:** Roberto Chiarelli, Maria Carmela Roccheri

**Affiliations:** Dipartimento di Scienze e Tecnologie Molecolari e Biomolecolari STEMBIO, Università di Palermo, Viale delle Scienze Edificio 16, Palermo 90128, Italy; Email: roberto.chiarelli@unipa.it

**Keywords:** autophagy, apoptosis, arsenic, cadmium, stress, sea urchin embryos

## Abstract

In recent years, research on the autophagic process has greatly increased, invading the fields of biology and medicine. Several markers of the autophagic process have been discovered and various strategies have been reported studying this molecular process in different biological systems in both physiological and stress conditions. Furthermore, mechanisms of metalloid- or heavy metal-induced toxicity continue to be of interest given the ubiquitous nature and distribution of these contaminants in the environment where they often play the role of pollutants of numerous organisms. The aim of this review is a critical analysis and correlation of knowledge of autophagic mechanisms studied under stress for the most common arsenic (As) and cadmium (Cd) compounds. In this review we report data obtained in different experimental models for each compound, highlighting similarities and/or differences in the activation of autophagic processes. A more detailed discussion will concern the activation of autophagy in Cd-exposed sea urchin embryo since it is a suitable model system that is very sensitive to environmental stress, and Cd is one of the most studied heavy metal inductors of stress and modulator of different factors such as: protein kinase and phosphatase, caspases, mitochondria, heat shock proteins, metallothioneins, transcription factors, reactive oxygen species, apoptosis and autophagy.

## 1. Introduction

### 1.1. Autophagy

Macro-autophagy, here after referred to as autophagy, is a major intracellular pathway for the degradation and recycling of cytosolic components, in both basal and stress conditions, in eukaryotic cells. Autophagy is a process of bulk degradation of toxic protein aggregates and damaged organelles, in which portions of the cytoplasm are sequestered into double-membrane vesicles known as autophagosomes, and then fuse with lysosomes to form single-membrane autolysosomes; ultimately, the contents of autolysosomes are degraded by lysosomal hydrolases and recycled for energy utilization. In this context, autophagy is involved in cell survival [1]. The execution and regulation of the autophagic program occurs by the expression of several autophagy-related (Atg) genes, having a high degree of conservation among species, from yeast to human. Depending on the environmental stress and cell type, this pathway acts as either a survival or death safeguard mechanism. However, massive and persistent autophagy can kill severely damaged cells by a caspase-independent form of cell death, termed type II cell death [2,3].

Similarly to apoptotic programmed cell death, autophagy is an essential part of embryo development, growth regulation and maintenance of homeostasis in multicellular organisms. Autophagy is critical to the process of embryo development because some animal models have shown that the lack of autophagy leads to arrest, delays or defects in development [4].

Autophagy responds to the cellular environment, and therefore any alteration in the environment may lead to the dysregulation of this process, potentially resulting in cell death.

The autophagic process occurs constitutively at basal levels and appears to be activated as an adaptive response to a variety of intracellular and extracellular stimuli, including nutrient deprivation (starvation), hormonal treatments, bacterial or viral infections, accumulation of misfolded proteins and damaged organelles, toxic stimuli, radiation and many agents of stress. In general, it seems that autophagy is necessary for cell survival under stress through removal of damaged proteins and organelles. There is a potential link between autophagy and a number of human diseases, such as cancer, cardiomyopathy, and neurodegenerative disorders, including Alzheimer’s, Parkinson’s, and Huntington’s.

Furthermore, autophagy may also be a strategy for self-destruction through the induction of programmed cell death (PCD), which is different from apoptosis. PCD is an essential and highly orchestrated process, which plays an important role in the development, cellular homeostasis, and prevention of cancer cell growth. The two main PCD types are referred to as apoptosis (PCD I) and autophagy (PCD II) [5].

There is emerging evidence that autophagy plays a critical role in the generation of antineoplastic responses [6,7]. Depending on the cellular context, autophagy may act as a protective mechanism for malignant cells or exhibit opposing effects and promote antineoplastic responses [8,9,10]. In fact, tumor growth in response to either blocked or induced autophagy has been described [11]. Some evidence suggests that autophagy may have an anticancer role: The autophagy gene beclin-1 acts as a tumor suppressor in mice and is monoallelically deleted in 40–75% of human breast, ovarian and prostate tumors [12,13]. Furthermore, the established tumor suppressor gene p53 induces autophagy [14,15] whereas the oncogenic proteins Bcl-2 and Bcl-XL interact with Beclin-1 to inhibit autophagy [16]. However, in certain circumstances, Bcl-2/Bcl-xL may have opposite effects on autophagy, depending on whether they are associated with mitochondria or the ER [17]. In addition, AKT activation (which is increased in many cancers) leads to increased mTOR activation and blockage of autophagy. Indeed, rapamycin and its derivatives inhibit mTOR activation, induce autophagic cell death and are under clinical investigation as anti-cancer compounds [18].

On the other hand, there is evidence that defects in autophagic responses are associated with tumorigenesis [19], whereas various inductors of autophagy exhibit tumor suppressor activities [20,21].

Autophagy is considered one of the most important molecular responses against different environmental stress agents and can be considered as a biomarker of toxicants. More knowledge regarding this process could provide valuable toxicological data for hazard and risk assessment.

In this review we considered the effect of some non-essential heavy metals in different model systems correlated to autophagy. 

### 1.2. Heavy Metals and Metalloids

The environment is composed of the atmosphere, earth and water. According to the World Health Organization, more than 100,000 chemicals are released into the global environment every year as a consequence of their production, use and disposal. The fate of a chemical substance depends on its chemical application and physical-chemical properties, in combination with the characteristics of the environment where it is released. Chemical substances or contaminants discharged into the environment may be “natural”, a substance that can occur without human introduction, or *vice* *versa* “manmade”. The chemicals, both from natural and anthropogenic sources, are of considerable interest based on their ability to induce the activation of defense systems or interrupt the developmental program. Among these substances we consider the heavy metals. The term heavy metal refers to any metallic chemical element that has a relatively high density and is toxic or poisonous at low concentrations. 

Approximately 30 metals and metalloids are potentially toxic to humans; examples include mercury (Hg), cadmium (Cd), chromium (Cr), lead (Pb), and arsenic (As). As trace elements, some heavy metals (e.g., copper, selenium, zinc) are essential to maintain the metabolism of organisms; however, at higher concentrations, they can lead to poisoning.

Heavy metals, as they are not biodegradable and persistent in the environment for long periods, cause serious eco-toxicological problems. In addition, some toxic metals may mimic essential metals and thereby gain access to important molecular targets. To a small extent they enter into organisms via food, drinking water and air and are bio-persistent pollutants that accumulate at the top of the food chain [22]. Heavy metals can enter a water supply by industrial and consumer waste, or even from acidic rain breaking down soils and releasing heavy metals into streams, lakes, rivers, and groundwater.

Heavy metals and metalloids are dangerous because they tend to bioaccumulate. Bioaccumulation means an increase in the concentration of a chemical in a biological organism over time compared to its concentration in the environment. Compounds accumulate in living things any time they are taken up and stored faster than they are broken down (metabolized) or excreted.

Compounds of heavy metals and metalloids are known to be stress agents and in some cases, as well as inducing apoptosis, are able to trigger autophagy.

## 2. The Effects of Heavy Metals and Metalloids on Cells

### 2.1. Arsenic

Arsenic (As) is a metalloid that is rarely found as a free element in the natural environment. It is abundant in the Earth’s crust and is variously distributed in soils, detectable in many waters, and in almost all tissues of animals and plants. A substantial amount of As, in various chemical forms and in various oxidation states, may be present in the environment both based on the effect of erosion processes and as a consequence of production by human activities. As has two oxidative states: a trivalent form and a pentavalent form, moreover, in is found in the form of arsenous acid (H_3_AsO_3_) and its salts, and arsenic acid (H_3_AsO_5_) and its salts.

As compounds are well-known toxic and carcinogenic agents, which, depending on oxidation state and chemical species, cell type, exposure concentrations, and time, can induce apoptosis. Because As is an ubiquitous contaminant, organisms are constantly exposed to this metal. The toxic effects of As that are of most concern to humans are those that occur from chronic, low-level exposure, and are associated with a range of human diseases, including various internal cancers. The genotoxic and co-genotoxic effects of inorganic arsenicals are well documented in mammalian systems, both *in* *vitro* [23] and *in* *vivo* [24].

Arsenic trioxide (As_2_O_3_) has a long history of use as a pharmaceutical agent for its antitumor properties. However, As_2_O_3_ has had a mainly unfavorable reputation due to its poisonous or environmental toxicity. Recently, As_2_O_3_ has shown considerable efficacy in treating patients with acute promyelocytic leukemia (APL). As_2_O_3_ activates numerous intracellular signal transduction pathways (including ROS, mitochondrial disruption, caspase activation, p53, and the MAPK signaling pathway), resulting in induction of apoptosis, promotion of differentiation, or inhibition of angiogenesis [25,26].

Although much is known about the mechanisms by which the arsenic compounds induce apoptosis, very little is known about the potential involvement of autophagy as a regulator of As-dependent neoplastic cell death. Recent studies have shown that it could cause autophagic cell death in malignant cells, including leukemia and malignant glioma cells.

As_2_O_3_ has important anti-leukemic effects *in* *vitro* and *in* *vivo* and is known to mediate its effects via its ability to induce apoptosis. Current research has revealed that autophagy can be an alternative or accompanying process to apoptosis in As-exposed cells. Goussetis *et* *al.*, [27] examined the capability of As_2_O_3_ to induce autophagic cell death in leukemic cell lines: Their data demonstrate that As is a potent inducer of autophagy, but not apoptosis [28], and such an induction is mediated by engagement of the MEK/ERK pathway. Interestingly, it has been demonstrated that a treatment of acute promyelocytic leukemia with As_2_O_3_ is one of the most successful examples of targeted cancer therapy that is being used in the clinic at present. This treatment causes proteolytic degradation of a highly oncogenic protein, the PML/RARA fusion protein that sustains malignant transformation supporting concomitant disease remission. Autophagy appears to regulate the basal turnover of PML/RARA: Autophagy-mediated suppression of this oncoprotein may represent a tumor-suppressor mechanism that prevents or delays the onset of this type of cancer [29]. In the human T-lymphocytic leukemia cell line Molt-4, As_2_O_3_ induces autophagy through the up-regulation of Beclin-1 (a 60-kDa coiled-coil protein essential for autophagosome formation), as an additional mechanism to apoptosis [30].

In HL-60, an acute promyelocytic leukemia cell line, several groups have established the induction of apoptosis by As through mechanisms that may be initiated by reactive oxygen species (ROS) generation as well as direct As targeting of the mitochondrial permeability transition pore [31,32]. Morphological characteristics of apoptosis (membrane blebbing, chromatin condensation) in HL-60 cells exposed to several As species, with particular potency in the trivalent arsenicals were documented [33,34]. On the other hand, a study by Yang *et* *al.*, [35] have provided evidence that arsenic trioxide-exposed HL-60 cells undergo cytotoxicity that is characterized by apoptosis, but also concurrently by autophagy. The data suggest that autophagy has differential effects on the As_2_O_3_-induced death of HL60 cells: It may be a protective mechanism against apoptosis in the earlier period of As_2_O_3_ treatment, while it promotes apoptosis and/or leads to autophagic death in the later period of As_2_O_3_ treatment.

Autophagy that regulates cell survival may act through interference with the mitochondria-mediated apoptotic pathway. It is believed that the antitumor efficacy of As involves the inhibition of proliferation and the triggering of apoptosis in tumor cells, but the relationship between autophagy and apoptosis and its antitumor effects still remain obscure. The process of autophagy could lead to “mitochondrial quality control”: Prevention of oxidative damage and mutagenesis through the removal of damaged mitochondria, which are a major source of toxic ROS [36]. Some investigators have shown that ROS regulate autophagy through several different mechanisms, including up-regulation of Beclin-1, oxidation of Atg4 and by causing mitochondrial dysfunction [37]. Since both ROS and autophagy are involved in cancer initiation and progression, it is thus essential to consider the regulation of ROS-induced autophagy in the development of cancer therapeutics [38]. Hence it is possible that autophagy may be an alternative process for the generation of the antitumor effects in other malignant cell types. 

It has been shown that in human malignant glioma cells, the most common and lethal tumor in the central nervous system, As induces autophagic cell death but not apoptosis. Specifically, cell death included the involvement of autophagy-specific marker LC3 (microtubule-associated protein light chain 3) and was accompanied by damage of mitochondrial membrane integrity, but not by caspase activation. In that case a novel mechanism has been implicated, suggesting the possibility that As_2_O_3_ may induce a hypoxic state in tumor cells and subsequently, through up-regulation of a mitochondrial cell death protein (BNIP3), lead to autophagic cell death [28]. But in recent years it has become evident that the up-regulation of autophagy is often wrongfully described as an alternative cell death program [39]. Therefore autophagy may, as we reported, also serve as a pro-survival mechanism and its up-regulation therefore reflects the cell’s attempt to counteract death signaling.

Recently, it has also been shown that arsenic trioxide induces a Beclin-1 independent autophagic pathway in ovarian carcinoma cells [40]. In fact, these authors observed that As_2_O_3_ alters the expression of TGFβ signaling mediators via the generation of reactive oxygen species (ROS). Their results suggest that the increase in SnoN (a TGFβ signaling mediator) expression leads to changes in LC3-II levels, implicating a role for SnoN in As_2_O_3_-induced autophagy, which could potentially bypass the apoptotic pathway. SnoN can be considered a novel therapeutic target for ovarian cancers.

Human lymphoblastoid cell lines (LCL) have been used as a model system in arsenic toxicology for many years, but the exact mechanism of As-induced cytotoxicity in LCL is still unknown. Bolt *et* *al.*, [41] investigated the cytotoxicity of sodium arsenite in LCL 18564 using a set of complementary markers for cell death pathways. Markers indicative of apoptosis (phosphatidylserine externalization, PARP cleavage, and sensitivity to caspase inhibition) were uniformly negative in arsenite-exposed cells. Interestingly, electron microscopy, acidic vesicle fluorescence, and expression of LC3 protein in LCL identified autophagy as an arsenite-induced process that was associated with cytotoxicity. Autophagy appeared to be the predominant process in LCL cytotoxicity induced by arsenite. It was unclear, however, whether LCL autophagy was an effector mechanism of arsenite cytotoxicity or alternatively a cellular compensatory mechanism. For this reason the authors, in a subsequent study, characterized As-induced effects in LCL cultures derived from seven human donors exposed *in* *vitro* to levels of sodium arsenite (0.75 µM) commonly encountered in the environment, and hence minimally cytotoxic. As-exposure resulted in inhibition of cellular growth and induction of autophagy, measured by the expansion of acidic vesicles, over the eight-day exposure duration. Gene expression analysis revealed that As-exposure increased global lysosomal gene expression, which was associated with increased functional activity of the lysosome protease, cathepsin D [42]. This is the first report of As-exposure modulating the regulation of genes encoding lysosomal constituents. The lysosome, historically considered a dead end for damaged cellular contents, is emerging as a critical player that actively maintains cellular homeostasis.

Tetraarsenic hexoxide (As_4_O_6_) has been used as anticancer in U937 human leukemic cells: The growth of U937 cells was inhibited by this treatment in a dose- and a time-dependent manner. This study suggests that As_4_O_6_ should induce Beclin-1-induced autophagic cell death as well as caspase-dependent apoptosis and that it might be a promising agent for the treatment of leukemia [43]. A comparison study of the anticancer effects between As_2_O_3_ and As_4_O_6_ demonstrated that As_4_O_6_ was more effective in suppressing human cancer cells *in* *vitro* and *in* *vivo*, and that the As_4_O_6_-induced cell death pathway was different from that of As_2_O_3_[44].

A study by Huang *et* *al.*, [45] provides the first evidence that the human uroepithelial cells *in* *vitro* respond directly, within 48 h of exposition to sodium arsenite, by promoting extensive vacuolation. They suggest that As induces cell death not only via apoptosis but also via autophagy, increasing LC3B and Beclin-1 protein expression, maybe via the ERK signaling pathway.

### 2.2. Arsenic in Combination with Other Heavy Metals or Radiation

Arsenic trioxide has also been used in combination with other treatments, such as ionizing radiation or heavy metals. Chiu *et* *al.*, [46,47] investigating the anticancer effects of ionizing radiation combined with arsenic trioxide in human malignant glioma cells and in human fibrosarcoma cells, *in* *vitro* and *in* *vivo*, found that this combined treatment increased cell death, inducing autophagy and apoptosis, compared to individual treatments. Furthermore, these researchers discovered that the combined treatment was affected by the inhibition of PI3K/AKT and the activation of ERK1/2, signaling pathways involved in regulating autophagy. Conversely, Larson *et* *al.*, [48] found that large alterations in the expression of Beclin-1 and associated proteins (essential effectors of autophagy) did not occur when human urothelial cells were malignantly transformed with, or exposed to, either Cd_2_+ or As_3_+.

Representative data from experiments performed in different model systems are reported in Table 1. 

**Table 1:** Arsenic concentration and induced cellular response with respect to autophagy.

As compounds (concentrations)	Autophagic effects	Experimental model	References
As_2_O_3_ (2 µM)	autophagic cell death, by activation of the MEK/ERK pathway; antileukemic effects	human leukemia cells	[27]
As_2_O_3_ (0,625–20 μM)	autophagic cell survival, in the earlier period of treatment; apoptosis and/or autophagic cell death, in the later period of treatment	HL60 leukemia cells	[35]
As_2_O_3_ (1 µM)	autophagy as clearance mechanism of the fusion protein PML/RARA	human leukemia cells	[29]
As_2_O_3_ (4 µM)	autophagic cell death, by up-regulation of Beclin-1, as well as apoptosis	human leukemia cells	[30]
As_4_O_6_ (0.5–3 µM)	autophagic cell death, by up-regulation of Beclin-1, as well as apoptosis, by caspase activation	U937 human leukemia cells	[43]
As_2_O_3_ (1–4 µM)	autophagic cell death, by up-regulation of BNIP3 and ERK 1/2, down-regulation of PI3K/AKT; antitumor effects	human malignant glioma cells	[28,46]
NaAsO_2_ (1–10 µM)	autophagic cell death, including increased levels of LC3B and Beclin-1, as well as apoptosis	human uroepithelial cells	[45]
NaAsO_2_ (6 µM)	autophagic cell death, including increased levels of LC3-II and autophagosomes/autolysosomes, not associated with apoptosis	human lymphoblastoid cells	[41,42]

### 2.3. Cadmium

Cadmium (Cd), commonly detected in aquatic and terrestrial environments, is a heavy metal released both from natural sources (e.g., volcanism, erosion) and anthropogenic activities (e.g., pigments, nickel–cadmium batteries, smelting and refining of metals and many other sources). The presence in the environment of this metal has increased because of its large utilization in some industrial and agricultural activities. Cd is a potent cell poison that causes different types of damage including cell death, and is a highly toxic environmental pollutant. 

The toxicity associated to Cd for living organisms, even at low concentrations, is amplified as a consequence of the long biological half-life of the metal. The metal is highly dangerous not only because it easily penetrates the cells via transport mechanisms normally used for other purposes, but also because it is eliminated very slowly, as it is not prone to bacterial degradation or detoxification.

Since Cd is a non-essential metal, which is not physiologically present in organisms, it is irreversibly accumulated in cells, interacting with cellular components and molecular targets. Cd toxicity has been associated with: blockage of oxidative phosphorylation, glutathione depletion and inhibition of antioxidant enzymatic activity, production of ROS, DNA damage and inhibition of relative repair mechanisms, and also to general reduction of protein synthesis coupled to an increase in stress proteins (HSPs) [49].

Cd has toxic effects on the lungs, kidneys, liver, and immune system. Furthermore the occurrence of apoptotic events after Cd exposure has been demonstrated in numerous organisms and cell lines. Human exposure occurs by inhalation (cigarette smoke) and by ingestion of Cd-contaminated food or water.

Cd also acts as a cancer promoter that causes transformation in cultured cell lines [50] and produces malignant tumors in testes, prostate, lungs, pancreas and liver of experimental animals. In addition to effects on gene expression and DNA repair, Cd carcinogenesis probably involves inhibition of apoptosis [51]. In any case, the outcome for a cell exposed to Cd is dependent not only upon the duration and level of exposure, but also on other factors intrinsic to the cell itself, and its current metabolic state.

Some of the pathways leading to cell death and survival in mesangial cells are summarized in a review by Templeton *et* *al.*, [52]. The authors report that the effects of Cd depend on the concentration levels in exposed cells: Low concentrations (0.5 µM CdCl_2_) lead to proliferation or delayed apoptosis, intermediate concentrations (10 µM CdCl_2_) can induce autophagy and/or cause various types of apoptotic death, and only very high concentrations (>50 µM CdCl_2_) provoke necrosis.

On the other hand, a recent study by Yang *et* *al.*, [53] shows that Cd treatment induces death in mesangial cells, leading to nephrotoxicity via multiple pathways, including the ROS-GSK-3β autophagy, calcium-ERK autophagy and apoptosis, and calcium-mitochondria-caspase apoptosis pathways. In the ROS-GSK-3β autophagy pathway, Cd might act on mitochondria to generate ROS, which in turn results in activation of GSK-3β leading to autophagic cell death. In the calcium-ERK autophagy and apoptosis pathway, Cd acts on the endoplasmic reticulum to induce elevation of the cytosolic calcium concentration, which in turn activates ERK leading predominantly to autophagic cell death and a minor level of apoptotic cell death. In the calcium-mitochondria caspase apoptosis pathway, Cd induces the release of calcium by the endoplasmic reticulum and then calcium depolarizes the membrane potential of mitochondria, which in turn activates caspases 9 and 3 leading to apoptotic cell death.

Research by Wang *et* *al.*, [54] points in the same direction in that it reports that treatment of mesangial cells for 24 h in serum-depleted medium causes both autophagy and apoptosis, in a dose dependent manner. The authors also give a demonstration that Cd-induced autophagy is mediated directly by activation of GSK-3β (glycogen synthase kinase-3β) [55]. 

In human umbilical vein endothelial cells, low concentrations of cadmium nitrate (≤10 µM) could inhibit apoptosis induced by deprivation of serum and basic fibroblast growth factor, and promote autophagy, while apoptotic cell death was induced by higher concentrations of Cd (>20 µM). Findings support the notion that moderate levels of autophagy may prevent apoptosis, but excessive autophagic vacuolization also leads to cellular stress, even cell death [56].

Moreover, Cd induces autophagy, in a dose- and time-dependent manner, as demonstrated by the increase of LC3-II formation and the GFP-LC3 puncta cells in skin epidermal cells. Results suggest that Cd-mediated ROS generation causes PARP activation and energy depletion, and eventually induces autophagy through the activation of LKB1–AMPK signaling and the down-regulation of mTOR. But in this case, data reveal that Cd-induced autophagy predominantly leads to cytotoxicity and cell death [57].

Chargui *et* *al.*, [58] explored the *in* *vivo* early effects of Cd intoxication on rat renal proximal tubule cells (PCT) using sub-toxic Cd doses. They showed that low Cd concentrations and short exposures did not affect the tubular functions nor did they induce apoptosis. In the meantime, metal accumulates in lysosomes of PCT cells, where it triggers cell proliferation and autophagy, detected by increases of LC3-II and the presence of punctuate LC3. Accumulation of Cd within the lysosomes and increases in lysosomal numbers may reflect sequestration and detoxification of this metal. In this context, autophagy, by enhancing the cell’s tolerance, could provide an early adaptive mechanism avoiding apoptosis.

Conversely, one of the major causes of chronic kidney disease (CKD) is the continued exposure to low levels of Cd. One of the causative mechanisms of chronic kidney disease is thought to be oxidative stress, therefore the involvement of mitochondria is highly plausible given that these organelles are central to the formation of excess ROS and are known to be key intracellular targets for Cd. Autophagy may remove dysfunctional mitochondria, reducing the excessive production of ROS, but if sufficient mitochondria are removed in this way then the viability of the cell cannot be maintained [59]. With regards to Cd-nephrotoxicity, the role and implication of autophagy and autophagic cell death necessitate further definition [60].

#### 2.3.1. Cadmium in Combination with Chromium

Cadmium has also been used in combination with chromium (Cr), a heavy metal that depending on its concentration induces both acute poisoning and chronic toxic effects that may contribute to carcinogenesis and the induction of degenerative diseases.

It was recently reported that autophagy is implicated in the response of hematopoietic stem/progenitor cells (HSPC) to toxic concentrations of sodium chromate and cadmium chloride, highly soluble salts releasing hexavalent chromium (Cr[VI]) and cadmium (Cd) cations, respectively, two of the best known toxic and carcinogenic heavy metals [61,62]. Results suggest that cells exposed to sub-toxic (0.1 μM) and toxic (10 μM) concentrations of each of the two cations (co-treatment) show autophagic morphologies (as autophagosomes/autophagolysosomes) and do not show the morphological hallmarks of apoptosis, contrary to what was reported for similar treatments in myelogenous leukemia and promyelocytic leukemia cell lines as well as peripheral blood mononuclear cells [63].

Autophagy may lead to cell death if carried out excessively, but may also act as a temporary survival pathway in cells under stress, mitigating metal-induced toxicity, delaying or preventing apoptosis, contributing to the conservation of tissue renewal capability, perhaps because removal of damaged mitochondria may keep cells under the threshold of caspase activity needed to trigger death. It may be that in the hematopoietic lineage, autophagy and apoptosis are both involved in the response to Cr(VI)- and Cd-induced toxic stress, and the molecular switch between the two pathways could be regulated according to differentiation.

#### 2.3.2. Effects of Cadmium on Aquatic Invertebrates

Heavy metal persistence and accumulation in biota is widespread in the aquatic environment. Aquatic invertebrates are known to accumulate high levels of heavy metals in their tissues and yet survive in polluted environments. Their tolerance of high metal content in tissues probably depends on the ability of these organisms to regulate heavy metal cation concentrations inside the cell and to accumulate excess metal in non-toxic forms [64,65].

Sub-lethal effects of Cd on population growth rate, gametogenesis and embryogenesis have been described in a variety of aquatic invertebrate organisms, such as sponges, molluscs, crustaceans, echinoderms, some of which are considered as good bio-indicators to assess the contamination of the aquatic environment. Therefore, the effects of Cd on these organisms have been studied, examining: accumulation of metal in adult tissues, embryonic development perturbation, stress protein induction, expression of detoxification genes, apoptosis and related pathways [49].

There is increasing evidence of Cd accumulation in the digestive and excretory organs of some benthonic invertebrates. Very high concentrations of the metal were found in the digestive gland of a few species of Antarctic molluscs [66] and in the digestive gland and kidney of the mussel *Crenomytilus* *grayanus* [67], suggesting again that food is the primary pathway for Cd bio-accumulation and that the digestive gland plays a major role in the subsequent storage and detoxification. In the renal tissue of Antarctic bivalve *Laternula* *elliptica* the high levels of Cd and its bio-accumulation can be a probable advantage for environmental adaptation in the Antarctic marine environment [68]. 

Responses to Cd exposure are well documented for *Ruditapes* *decussatus*. In this bivalve mollusc, the metal is mainly accumulated in the gill following transport to the digestive gland, where it is continuously accumulated. A paper by Chora *et* *al.*, [69] shows that Cd exposure causes changes in protein expression in the gill and digestive gland. The tissues exhibited different protein expression due to their different functions in molluscs. An overall decrease of proteins was detected in both treated tissues. The authors suggest that lysosomal autophagy could explain this decrease of proteins and at the same time it could be considered a survival strategy, but there is no experimental evidence that the autophagic process actually occurs.

Recently it has been suggested that autophagy appears to be a common target for many environmental pollutants, as lysosomes accumulate many toxic metals and organic xenobiotics, which perturb normal function and damage the lysosomal membrane. 

At present, the study on the role of autophagy in aquatic organisms exposed to heavy metals is a new emerging research field.

#### 2.3.3. Stress Response in Cd-Exposed Sea Urchin Embryos

Sea urchins are an ancient group (at least 450 million years old) of Class Echinoidea in the Phylum Echinodermata, with hundreds of species known in the world’s oceans. Since about 1880, the eggs and sperm of sea urchins have been used for the study of fertilization, the metabolic activation of development and gene regulatory mechanisms governing embryogenesis. In addition, the genome of one sea urchin, *Strongylocentrotus* *purpuratus*, is known, and the size is ~800 Mb, compared to the human genome of ~3,200 Mb [70]. This aspect permitted to explore whether parts of the vertebrate toolkit are also present in invertebrate deuterostomes, allowing ascertaining a lineage-specific evolution of various molecular networks. 

This marine organism directly interacts with its environment and is susceptible to effects of several aquatic contaminants. Several molecular mechanisms can be adopted as a defense mechanism against any environmental chemical, physical and mechanical stress, in an attempt to preserve the developmental program. The sea urchin embryo represents a suitable model system to investigate the adaptive response of cells exposed to stress during development and differentiation [71,72].

Contrary to the view that embryos and larvae are the most fragile stages of life, development is stable under real-world conditions. Early cleavage embryos are prepared for environmental alterations by having high levels of cellular defenses already present in the egg before fertilization. Later in development, adaptive responses to the environment either buffer stress or produce alternative developmental phenotypes [73].

The chemicals of anthropogenic origin are of considerable interest for their ability to induce the activation of defense systems or interrupt the developmental program. Cd is a known stress agent and in some cases, as well as inducing apoptosis, is able to trigger autophagy. It is currently well accepted that autophagic cell death represents a separate route of programmed cell death significantly different from conventional apoptosis, but autophagy also represent a program of cell survival [74]. 

However, at present, there are few studies on the role of autophagy during development and on the putative protective function that it has in embryos exposed to stress. 

It was previously demonstrated that subacute/sublethal concentrations of Cd induce, during development of sea urchin embryos, morphological abnormalities, activation of specific stress proteins (HSPs), expression of metallothioneins and apoptosis, in a dose/time-dependent manner [75,76]. Essentially, Cd exerts its toxic action in the long term: Embryos accumulate the metal in the cells, as demonstrated by Atomic Absorption Spectrometry. It has been suggested that survival systems adopted during embryonic development of sea urchins can operate in tandem through a putative crosstalk [77,78]. 

#### 2.3.4. Analyzing Autophagy in Cd-Exposed Sea Urchin Embryos

Recently, autophagy activation in sea urchin embryos has been reported for the first time. The purpose of this study was to investigate whether autophagy occurs in *Paracentrotus* *lividus* embryos, a species of sea urchin very common in the Mediterranean Sea, both in physiological development and in response to stress, as a defense strategy after exposure to Cd, for the survival and maintenance of the developmental program.

Several experimental approaches were used to detect autophagy: identification of autophagolysosomes, by acidotropic dyes such as neutral red and acridine orange; immunodetection of LC3-II, by Western blot and immunofluorescence *in* *situ* analyses. 

**Figure 1:**
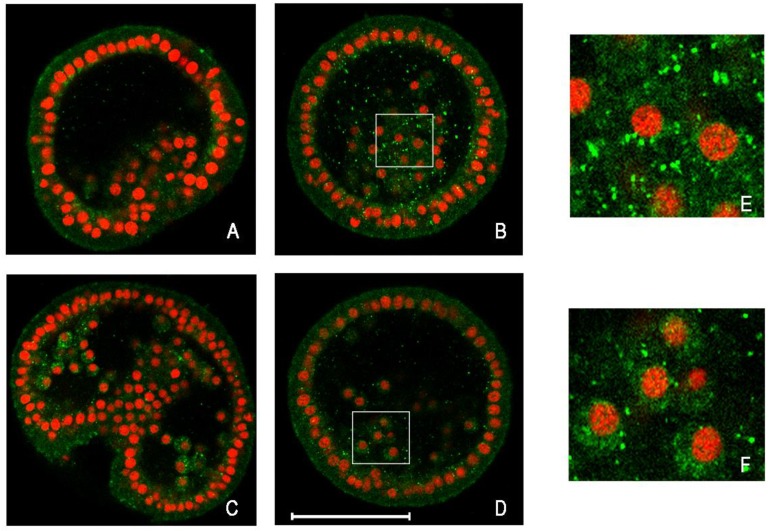
Detection by immunofluorescence of LC3 protein on whole-mount embryos of sea urchin. Equatorial optical sections captured by confocal laser scanning microscopy. In green, LC3 protein detection; in red, nuclei stained with propidium iodide. (**A**) Control embryo, after 18 h of growth; (**B**) Cd-treated embryo for 18 h; (**C**) Control embryo, after 24 h of growth; (**D**) Cd-treated embryo for 24 h; (**E**, **F**) Enlargements of a section of (**B**, **D**), respectively. Bar = 50 μm.

Results demonstrated that autophagy is a molecular process present in sea urchin embryos at a higher level after Cd treatment and at a basal level during physiological development. Specifically, the experiments revealed a higher level of autophagosomes for embryos treated for 18 hours with 1 mM CdCl_2_, compared with controls [74]. It should be noted that after 24 hours of treatment, embryos show a lower level of autophagosomes, probably because the apoptotic process becomes significant, as previously demonstrated [72,78]. In fact, immunodetection of LC3-II shows that it is mainly present in embryos treated for 18 hours rather than for 24 hours; in addition, the LC3 signal is largely cytosolic in control embryos, while it reveals a “punctuate” localization in embryos Cd-treated (Figure 1).

The results obtained suggest that probably the first defense strategies put in place by the embryo against stress by Cd, metallothioneins and HSPs, are able to produce a detoxifying and antioxidant effect that is not always sufficient to block the action of the toxic metal, depending on the extent of cell damage [79,80]. In such circumstances the mechanisms of programmed cell death, such as apoptosis, may be triggered [81]. 

**Figure 2:**
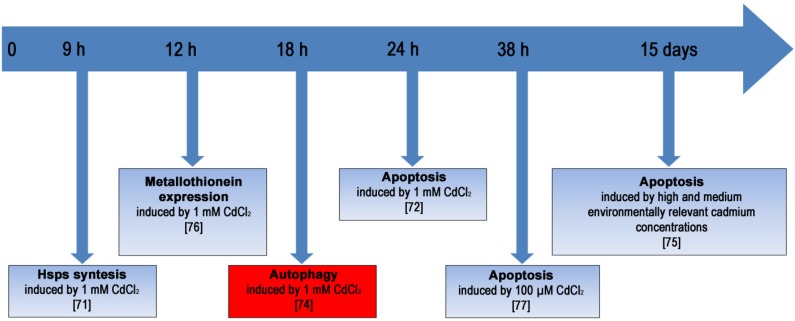
Diagram showing an overview of the most representative cellular, biochemical, and molecular events activated as defense strategies in embryos and larvae of *P.* *lividus* at different times post fertilization and various concentrations of CdCl_2_.

The high level of autophagy activation at a specific time seems to be critical because, although it is a normal mechanism of clearance, in some extreme cases it reaches high levels, indicating the possibility of modulating the activation and representing the high plasticity of this molecular mechanism for the survival of embryonic cells. Figure 2 shows a hypothetical model of reconstruction of the events that occur in embryos exposed to 1 mM CdCl_2_ for 24 hours, or exposed to lower Cd concentrations for longer periods of time. In this reconstruction, autophagy can be activated in a specific period of time, after HSPs and metallothionein induction and before apoptosis triggering, in a last attempt to safeguard the developmental program. However, the persistence of stress inevitably leads to apoptosis.

Sea urchin embryos continuously exposed to Cd provide a suitable model to study the role of autophagy in Cd-stress responses [82].

Representative data from experiments performed in different model systems are reported in Table 2.

**Table 2:** Cadmium concentration and induced cellular response with respect to autophagy.

Cd compounds (concentrations)	Autophagic effects	Experimental model	References
CdCl_2_ (3–24 µM)	calcium-mediated autophagy and apoptosis, through the ERK-dependent and mitochondria-caspase signaling pathways, respectively	mouse kidney mesangial cells	[54]
CdCl_2_ (1–10 µM)	autophagy that leads to cytotoxicity, as cell death mechanism; detected by an accumulation of autofagosomes and increased levels of LC3-II	mouse epidermal cell line	[57]
Cd (NO_3_)_2_ (1–10 µM) Cd (NO_3_)_2_ (>20 µM)	autophagy, as cell survival mechanism, detected by an accumulation of autolysosomes and increased levels of LC3-II;apoptotic cell death	human vascular endothelial cells	[56]
CdCl_2_ (0.3 mg/kg body mass/1, 3 and 5 days of intoxication )	autophagy, as cell survival mechanism	rat kidney	[58]
CdCl_2_ (1 mM for 18 hours of exposure) CdCl_2_ (1 mM for 24 hours of exposure)	autophagy as a survival mechanism detected by an accumulation of autolysosomes and increased levels of LC3-II;Apoptotic cell death	sea urchin embryos	[74]

## 3. Conclusions

Heavy metals and metalloids represent two of the most dangerous pollutants, and, due to their bioaccumulation, have been proven to be toxic both for human and environmental health. The adaptation to handle stress by metals through removal and recycling of damaged proteins and organelles may have been critical throughout evolution in biota naturally exposed to fluctuating levels of these toxicants [61]. 

Great progress has been made in recent years in understanding various facets of autophagy but there is still much need to understand the effects between exposure to various environmental factors and autophagy. This review shows that exposition to As and Cd compounds induces changes in autophagic response that are differently associated with specific disease. Identification of critical exposure to these compounds, related to the activation of autophagy, could help to identify analogous pathways triggered by potential useful drugs in therapy. 

The research summarized herein shows that autophagy and apoptosis may be used as an alternative and/or combined method by cells exposed to toxic concentrations of heavy metals or metalloids. However autophagy is also a process of cell survival that confronts the cell with the choice to live or to die. One possibility is that autophagy could represent a cell survival mechanism where cellular damage is not too extensive, or may lead to cell death wher the damage/stress is irreversible; in this last case it acts in association with apoptosis or with an independent pathway.

The relationship between autophagy and apoptosis and the antitumor effects still remain obscure. The multifaceted relation of autophagy with tumorigenesis and studies supporting a role for autophagy in both tumor-suppression and tumor-progression are still needed. However it is undeniable that the use of autophagy induction for cancer treatment presents novel therapeutic opportunities. As discussed above, induction of autophagic cell death by arsenic trioxide provides promising new opportunities for the treatment of resistant malignant gliomas, and it is now in clinical trials. The main challenge today is to determine when to apply each drug, depending on the biological understanding of the status of the cancer cells [7]. 

With regard to Cd-exposed cells, the role of autophagy in influencing cell survival or death is controversial and dependent on the exposure conditions. It was reported that autophagy did not protect cells from Cd-induced toxicity [54], but a recent investigation supported a protective effect of autophagy on Cd-induced cell death [83]. In fact, as observed for Cd stress, depending on the concentration and exposure time, this metal may induce cell survival (for lower expositions) or apoptosis (for elevated expositions).

Given the relevance of the tumor suppressor role for autophagy, an understanding of the mechanisms regulating the crosstalk with cell proliferation and differentiation both in embryogenesis and adulthood is an essential goal for researchers. 

It is to be highlighted, owing to the importance of autophagy in the turnover of cellular components, that this process is expected to have a crucial role in the events occurring during embryogenesis. Developing cells, in fact, have to adapt constantly and quickly to both intrinsic and environmental changes in order to survive and to differentiate. During this process, the damaged and unnecessary organelles need to be promptly cleared and the cell shape needs to be modified to adapt to new functions. The exact mechanisms by which autophagy contributes to embryonic phenotypes in multicellular eukaryotes are not clear. However, both in invertebrate and vertebrate organisms, it is generally thought that autophagy plays an essential dual role both in the adaptation to stress and starvation phenomena occurring during morphogenesis and in cell elimination, in concert with the apoptotic machinery [84]. In addition, autophagic cell death appears to precede apoptosis when massive cell elimination is required during development. To fully understand the molecular responses induced by heavy metals or metalloids it is appropriate to incorporate new biomarkers with high sensitivity and speciﬁcity, and autophagy seems be a good marker of toxicity since it is a specific mechanism conserved from yeast to humans. 

In this contest, the sea urchin embryo is one of the most suitable organisms to extend the observations on autophagy made in budding yeast, plants, and mammalian cells and to ask questions about the control of autophagy both during physiological development and in stress condition, as we recently have reported in the guidelines for the use and interpretation of assays for monitoring autophagy [82]. 

In conclusion, autophagy may represent a key evolutionarily conserved response to toxic metals/metalloids. The relationships between autophagy and apoptosis and their molecular regulation, need to be explored in connection with exposure to specific toxicants. Although autophagy is aimed primarily at cell survival or at cell death, it is possible to remain a subject of debate for some time, for the immediate future the therapeutic uses of autophagy inducers and inhibitors in modulating both its survival and death-promoting properties show promise and continue to be actively explored. On the other hand, it may enhance cell death by a variety of mechanisms, thus leading to tissue pathology and organ damage. In either case, the outcome for a cell exposed to toxicants is dependent not only upon the duration and level of exposure, but also on other factors intrinsic to the cell itself, and its current metabolic state.

Finally, autophagy is one of a wide range of strategies that can be activated in response to heavy metal and metalloid toxicity.
